# Atypical Structure Revealed In Carbohydrate Deacetylase Unique to Bacteroides

**DOI:** 10.1063/4.0001075

**Published:** 2025-10-27

**Authors:** Krystle J McLaughlin, Lilith A Schwartz, Jordan O Norman, Sharika Hasan, Olive E Adamek, Elisa Dzuong, Jasmine C Lowenstein, Olivia G Yost, Banumathi Sankaran

**Affiliations:** 1 Vassar College, Poughkeepsie, NY, USA; 2 Lawrence Berkeley National Laboratory, Berkeley, CA, USA

## Abstract

*Bacteroides ovatus* is one of the most commonly found Bacteroides species in the human gut and has been linked to benefits like the suppression of intestinal inflammation. Conversely, increased populations of *B. ovatus* is correlated with several autoimmune disorders, including irritable bowel disorder (IBD). Bacterial cell surface carbohydrates such as capsular polysaccharides (CPS) may play a role in these host-microbe interactions as they have known immunomodulatory effects such as polysaccharide A (PSA) from *B. fragilis*. Despite their importance, few enzymes encoded in CPS biosynthetic loci have been studied. We report structural characterization of a putative polysaccharide deacetylase from *Bacteroides ovatus* (*Bo*PDA) encoded in a CPS biosynthetic locus. Four high resolution crystal structures (1.36 Å – 1.56 Å) of the enzyme bound to divalent cations Co^2+^, Ni^2+^, Cu^2+^ or Zn^2+^ revealed an atypical domain architecture that is unique to this enzyme. *Bo*PDA has a carbohydrate esterase 4 (CE4) superfamily catalytic NodB homology domain inserted into a carbohydrate binding module (CBM). *Bo*PDA also lacks the canonical CE4 His-His-Asp metal binding motif, instead using a non-canonical His-Asp dyad. We also performed carbohydrate binding and deacetylase activity assays to probe the function of the enzyme. Our work furthers understanding of this medically relevant microbe, as *Bo*PDA represents the first protein involved in CPS biosynthesis from *B. ovatus* to be characterized.

